# Penetrating keratoplasty: from aid dependence to independence

**DOI:** 10.22336/rjo.2017.24

**Published:** 2017

**Authors:** Mioara-Laura Macovei, Maria-Alexandra Nica

**Affiliations:** * Ophthalmology Department, “Dr. Carol Davila” Central Military Emergency University Hospital, Bucharest, Romania

**Keywords:** penetrating keratoplasty, corneal dystrophy, bullous keratopathy

## Abstract

The authors present five cases of bilateral corneal disorders, on which they performed penetrating keratoplasty. All five patients were clinically blind and after the surgery on one eye, they gained their visual independence. Visual acuity, intraocular pressure, fundus examination, and ultrasonography were used to evaluate the patients before and after the surgery.

## Introduction

The idea of restoring clarity to an opaque cornea has existed for far longer than the means to achieve it have been available. In 1905, Zirm performed the first successful human penetrating keratoplasty (PK) and thus the first successful human organ allograft [**1**].

Corneal blindness is the 4th cause of blindness globally [**2**]. Studies showed that visual loss is considered to have the strongest impact on the quality of life and it was included in the “worst thing that could happen” category [**3**]. Corneal transplantation has the best rate of survival and is the oldest and most frequent type of transplantation performed on humans [**4**]. Penetrating keratoplasty (PK) is a technique that allows the replacement of the central affected cornea with a healthy corneal graft from a donor. Nowadays, the advances in diagnosing certain disorders, the conservative surgical approach and changes in surgical practice, have influenced the indication for PK, which have been in a continuous change since 1940.

## Material and methods - Clinical cases

**Fig. 1:**
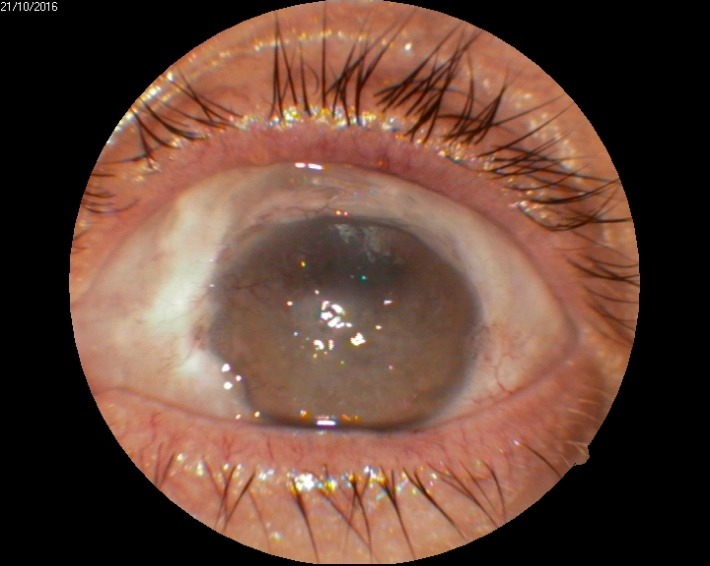
RE Bullous keratopathy

**Fig. 2:**
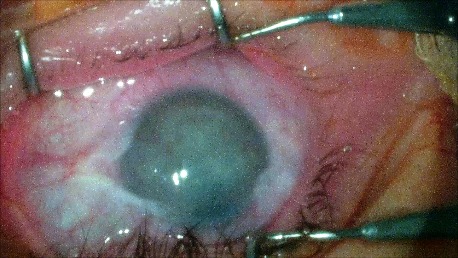
LE Bullous keratopathy preoperative

**Fig. 3:**
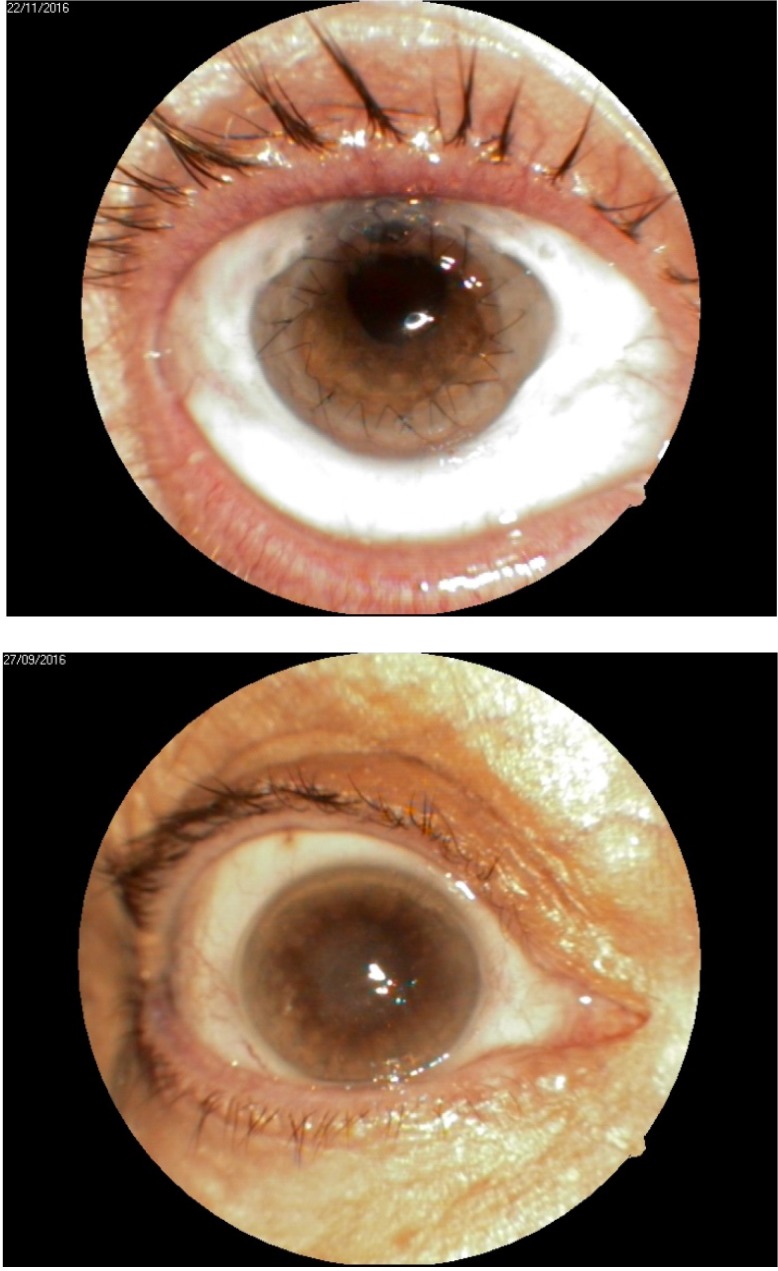
RE 7 months after PK and RE Corneal dystrophy

**Fig. 4:**
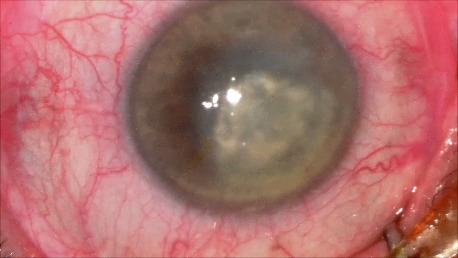
LE preoperative

**Fig. 5:**
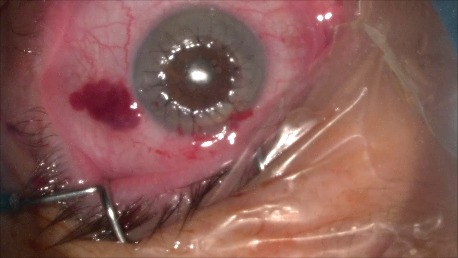
LE after PK

**Fig. 6:**
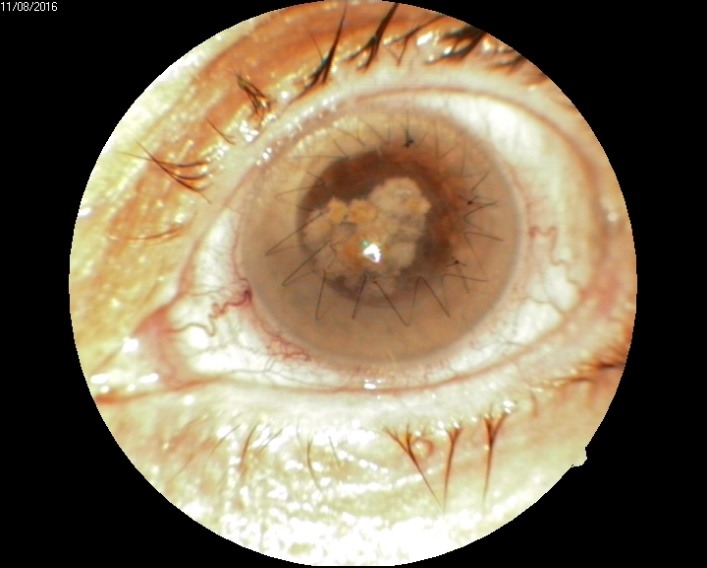
Band keratopathy 4 months after PK

**Fig. 7:**
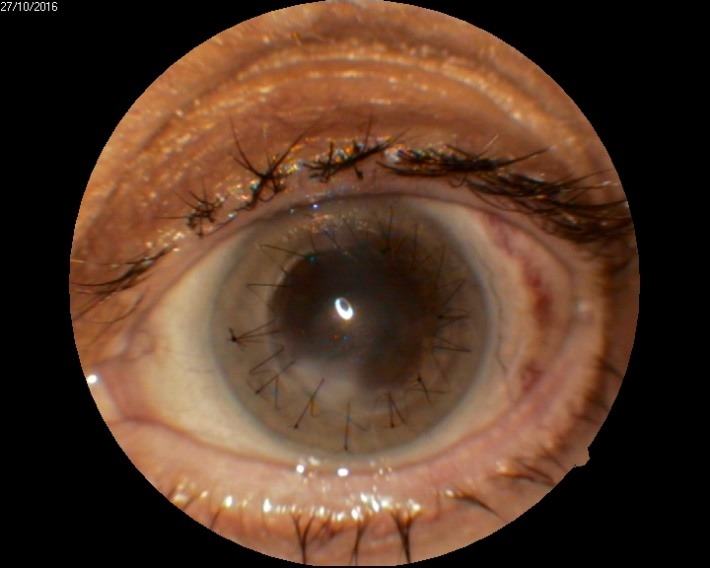
LE 5 months after PK

**Fig. 8:**
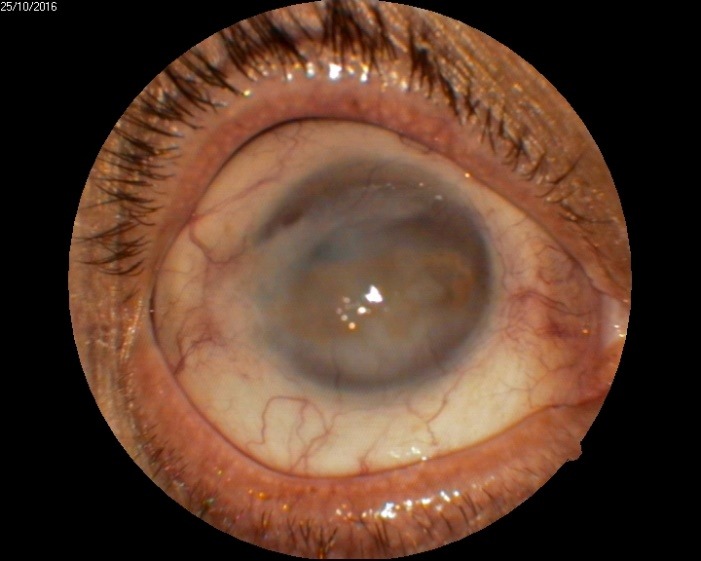
RE Phthisis bulbi

**Fig. 9:**
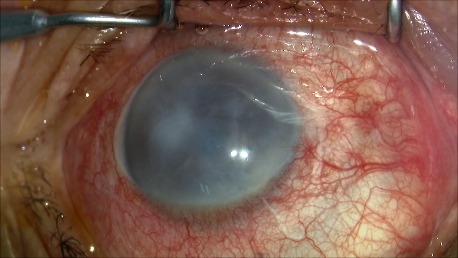
LE Bullous keratopathy

**Fig. 10:**
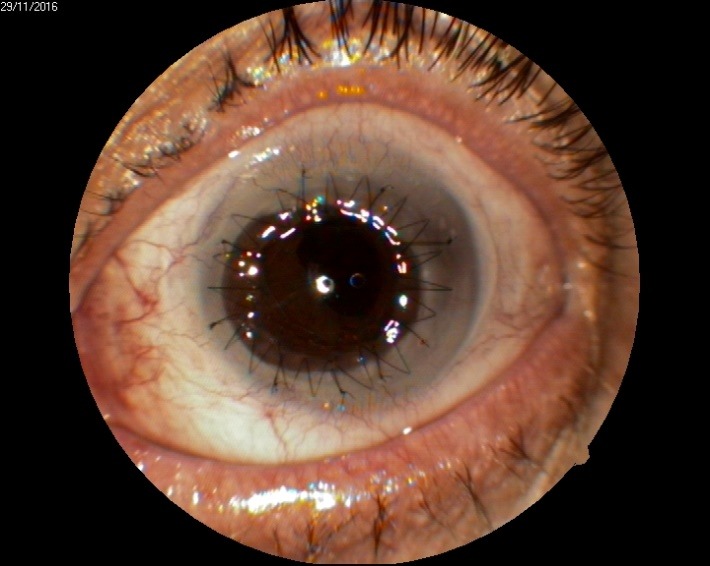
LE 4 months after PK

**Fig. 11:**
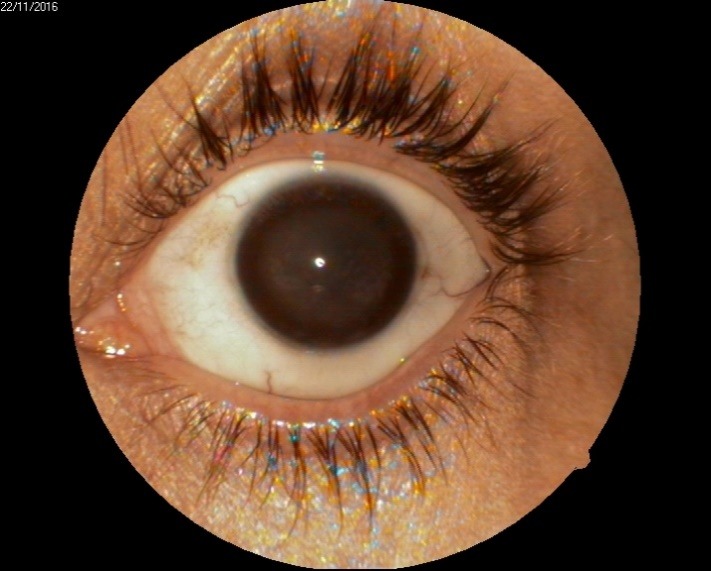
LE Macular corneal dystrophy

**Fig. 12:**
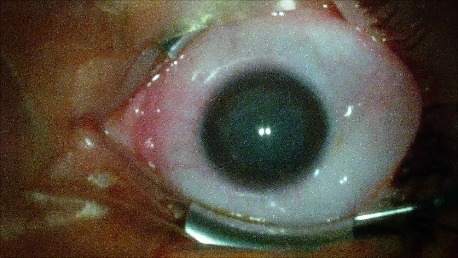
RE Macular corneal dystrophy

**Fig. 13:**
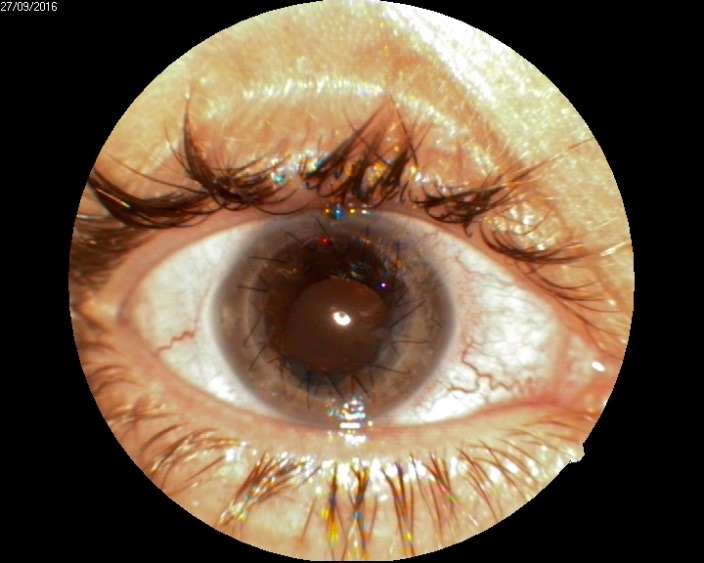
RE, 6 months after PK

**Fig. 14:**
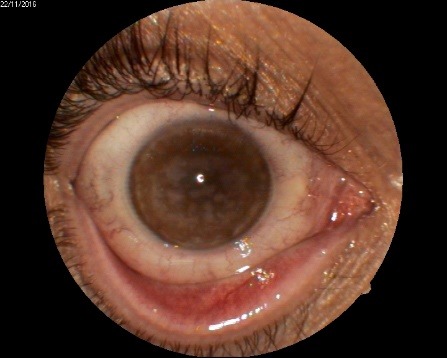
RE Corneal dystrophy

**Fig. 15:**
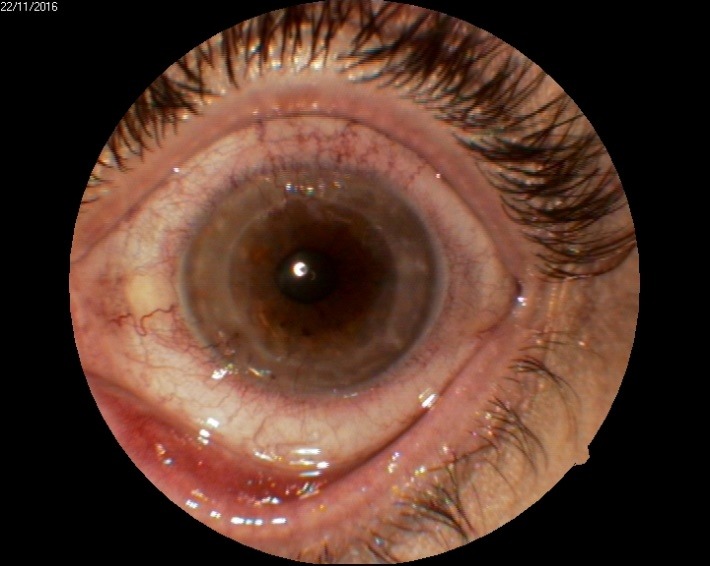
LE 1 year after PK

1. A 39-year-old patient diagnosed with BE: Pupillary pseudophakic - Fyodorov. Bullous keratopathy. Myopia. (**Fig. 1**,**2**). On clinical examination VA RE = HM (**Fig. 1**), VA LE = HM (**Fig. 2**), IOP BE - normal intraocular pressure (digital palpation) and A, B mode on ultrasonography: axial myopia, no other pathological changes. PK was performed on the RE, and, after 7 months, BCVA RE = 0,3 (**Fig. 3**).

2. A 86-year-old patient diagnosed with BE: Corneal dystrophy. Senile cataract. On clinical examination VA RE = HM (**Fig. 3**), VA LE = HM (**Fig. 4**), IOP BE - normal intraocular pressure (digital palpation) and A, B mode on ultrasonography: no pathological changes. After PK on the LE, BCVA LE = 0,3 (**Fig. 5**). Four months later, after PK, she presented with band keratopathy on the LE (**Fig. 6**) and her visual acuity decreased again to hand movement. We performed an excision of the superficial deposits, superficial lamellar keratectomy and finally we applied a thin layer of amniotic membrane. She came for her monthly check-up and her uncorrected vision was restored to 0.25 (**Fig. 7**).

3. A 72-year-old patient RE: history of penetrating trauma (phthisis bulbi). Lipid keratopathy. LE: pseudophakic eye (scleral-sutured IOL). Bullous keratopathy. Paracentral leukoma. Secondary glaucoma. On clinical examination VA RE = NLP (**Fig. 8**), VA LE = HM (**Fig. 9**), IOP LE - normal intraocular pressure (digital palpation), IOP RE - hypotonic globe and A, B mode on ultrasonography LE: no pathological changes, RE - disorganized globe. Four months after PK, her BCVA LE = 0.4 (**Fig. 10**).

4. Clinical case: A 37-year-old patient BE: Macular corneal dystrophy. On clinical examination VA LE = 0.1 (**Fig. 11**), VA RE = 0.08 (**Fig. 12**), IOP BE - normal intraocular pressure (digital palpation), A, B mode on ultrasonography: no pathological changes. Six months after PK, her BCVA RE - 0,4 (**Fig. 13**).

5. A 38-year-old patient BE: Macular corneal dystrophy. On clinical examination VA BE = 0.04 (**Fig. 14**), IOP BE - normal intraocular pressure (digital palpation), A, B mode on ultrasonography: no pathological changes. We performed PK on his left eye and five years after surgery his BCVA LE = 0.5 (**Fig. 15**).

## Conclusions

The purpose of PK in advanced and bilateral corneal disorders is determinant; this technique offers patients the ability to become independent of the family and society and physical, emotional, social, and economic rehabilitation, thus improving the quality of life. In Romania and in some other countries the only limitation in performing PK is the lack of corneal donors.

**Financial Disclosures**

The authors have no financial interests to disclose.
